# Arteriovenous fistula-related renal bleeding 5 days after percutaneous renal biopsy

**DOI:** 10.1007/s13730-019-00408-1

**Published:** 2019-06-18

**Authors:** Natsuki Shima, Noriko Hayami, Hiroki Mizuno, Masahiro Kawada, Akinari Sekine, Keiichi Sumida, Rikako Hiramatsu, Masayuki Yamanouchi, Eiko Hasegawa, Tatsuya Suwabe, Junichi Hoshino, Naoki Sawa, Kenmei Takaichi, Kenichi Ohashi, Takeshi Fujii, Seiji Minota, Yoshifumi Ubara

**Affiliations:** 1 Nephrology Center, Toranomon Hospital Kajigaya, 1-3-1, Takatsu, Kawasaki, Kanagawa 212-0015 Japan; 2 Department of Pathology, Toranomon Hospital, Tokyo, Japan; 3 Department of Pathology, Yokohama City University Hospital Graduate School of Medicine, Yokohama, Kanagawa Japan; 4 Okinaka Memorial Institute for Medical Research, Toranomon Hospital, Tokyo, Japan; 5 Division of Rheumatology and Clinical Immunology, Department of Medicine, Jichi Medical University, Shimotsuke, Tochigi Japan

**Keywords:** Arteriovenous fistula, Renal biopsy, Transcatheter arterial embolization, Macroscopic hematuria

## Abstract

A 32-year-old Japanese woman was admitted to our hospital for evaluation of microscopic hematuria with a positive family history. Percutaneous renal biopsy was performed under real-time ultrasound guidance using a 16-gauge automated needle and three specimens were obtained. She had no risk factors for hemorrhage. However, macroscopic hematuria developed from 5 days after biopsy and persisted for 4 days. Her Hb decreased markedly from 15.0 to 8.1 g/dL. Enhanced computed tomography revealed urinary tract hematoma, while the early arterial phase showed inflow of contrast medium into the left renal vein from a pseudoaneurysm on a branch left renal artery. Renal transcatheter arterial embolization was performed using platinum microcoils and the arteriovenous fistula was occluded. The patient did not require blood transfusion. Severe renal bleeding that causes urinary tract hematoma usually occurs within 24 h after renal biopsy, but the possibility of late-onset renal bleeding should be kept in mind.

## Introduction

Percutaneous renal biopsy (PRB) provides important information for assessing the diagnosis, management, and prognosis of patients with renal disease. Complications following renal biopsy have decreased thanks to improvements of imaging techniques and biopsy needles. Korbet et al. investigated complications of renal biopsy in patients with native kidneys. Death or major complications that required intervention, such as transfusion of blood products or invasive radiologic or surgical procedures, occurred in 6.6% of biopsies. Among these major complications, 57% were identified by 4 h after biopsy, while 72% were detected by 8 h, 89% by 24 h, and 11% after 24 h [1]. Simard-Meilleur et al. reported that complications developed in 84% of affected patients by 8 h, increasing to 86% at 12 h and 94% at 24 h [2]. Thus, major complications are considered to be uncommon from 24 h after PRB, but little has been reported about the occurrence of late complications from 24 h [3].

Here, we present a patient who developed arteriovenous fistula (AVF)-related macroscopic hematuria at 5 days after PRB. Transcatheter renal arterial embolization (TAE) was effective for control of renal bleeding.

## Case report

A 32-year-old Japanese woman was admitted to our hospital for evaluation of microscopic hematuria with a positive family history. Urinary occult blood had been detected during a health checkup at the age of 29 years, while 11–30 erythrocytes per high-power field (HPF) were seen in the urinary sediment at the age of 31 years. Her grandfather had required hemodialysis, but his renal disease was unknown.

On admission, the patient was 153.0 cm tall and weighed 44 kg, with a blood pressure of 108/75 mmHg, heart rate of 82/min, and temperature of 37.4 °C. There was no peripheral edema and no purpura, neuropathy, or arthritis.

Laboratory tests revealed that the white blood cell count was 5500/μL, hemoglobin (Hb) was 15.0 g/dL, and the platelet count was 29.9 × 10^4^/μL. In addition, serum albumin was 4.8 g/dL, blood urea nitrogen was 14 mg/dL, creatinine was 0.79 mg/dL, eGFR was 68.6 mL/min/1.73m^2^, aspartate aminotransferase (AST) was 19 IU/L, and alanine aminotransferase (ALT) was 21 IU/L, and γ-glutamyl transpeptidase was 18 IU/L. C-reactive protein was 0.0 mg/dL, while IgG was 961 mg/dL and IgA was 149 mg/dL (normal 110–410). Complement components C3 and C4 were 78 and 10 mg/dL, and CH50 was 37 U/mL (normal 30–50). Immunological tests including anti-double-stranded DNA antibody and antinuclear antibody suggesting autoimmune diseases were all negative. The activated partial thromboplastin time (APTT) was 29.5 s (normal 27–40) and the prothrombin time (PT) was 106.5% (normal > 75). Urinary protein excretion was 0.02 g/day, and the sediment contained 11–30 erythrocytes per HPF and detected dysmorphic red blood cells. Computed tomography (CT) of the chest and abdomen showed that her kidneys were normal in size with no morphological abnormalities. Definite nut cracker phenomenon in CT was not detected before renal biopsy.

## Renal biopsy

**Fig. 1:**
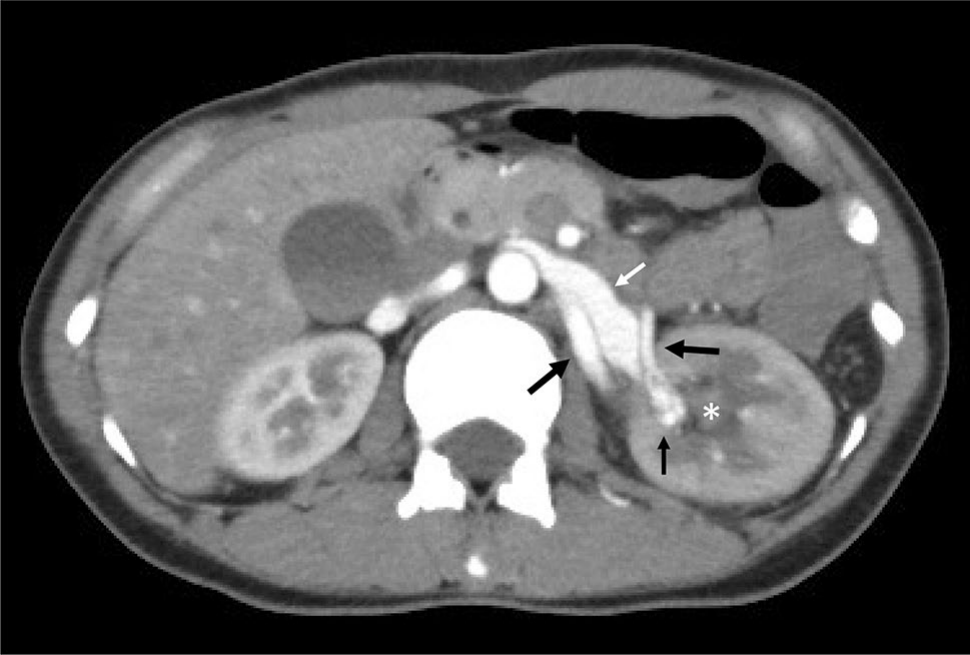
CT reveals urinary tract hematoma and inflow of contrast medium into the left renal vein from a pseudoaneurysm on a branch of the left renal artery in the early arterial phase. *Urinary tract hematoma, white arrow: dilated left renal vein, small black arrow: pseudoaneurysm or AVF, large black arrow: left renal artery and branches

Percutaneous renal biopsy was performed from the lower pole of the left kidney, using an automated device with 16 gauge and 16 mm (the stroke length) needle with real-time ultrasound guidance and three specimens were obtained. We had placed a urethral catheter at the time of renal biopsy. Blood pressure was stable without vasoactive agents throughout renal biopsy. Macroscopic hematuria occurred soon after renal biopsy, but subsided within 1 h. Macroscopic hematuria completely disappeared the next day after renal biopsy. The patient stayed about 24 h in strict bed rest after renal biopsy. Her Hb value was 11.8 g/dL and 12.1 g/dL on 1 day and 3 days after renal biopsy. Post-biopsy examination by Doppler ultrasonography was not been carried out. We used carbazochrome sodium sulfonate hydrate and tranexamic acid per orally for 3 days after renal biopsy. The patient discharged 4 days after renal biopsy. She did not complain any subjective symptoms such as back pain. Five days after the biopsy procedure, she developed back pain and macroscopic hematuria. She did not have any risk factor contributing to renal bleeding such as exercise or the use of anticoagulant. Macroscopic hematuria persisted for 4 days and her Hb decreased to 8.1 g/dL. Enhanced CT revealed urinary tract hematoma and the early arterial phase showed inflow of contrast medium into the left renal vein from a pseudoaneurysm on a branch left renal artery (Fig. 1).

## Angiography

**Fig. 2:**
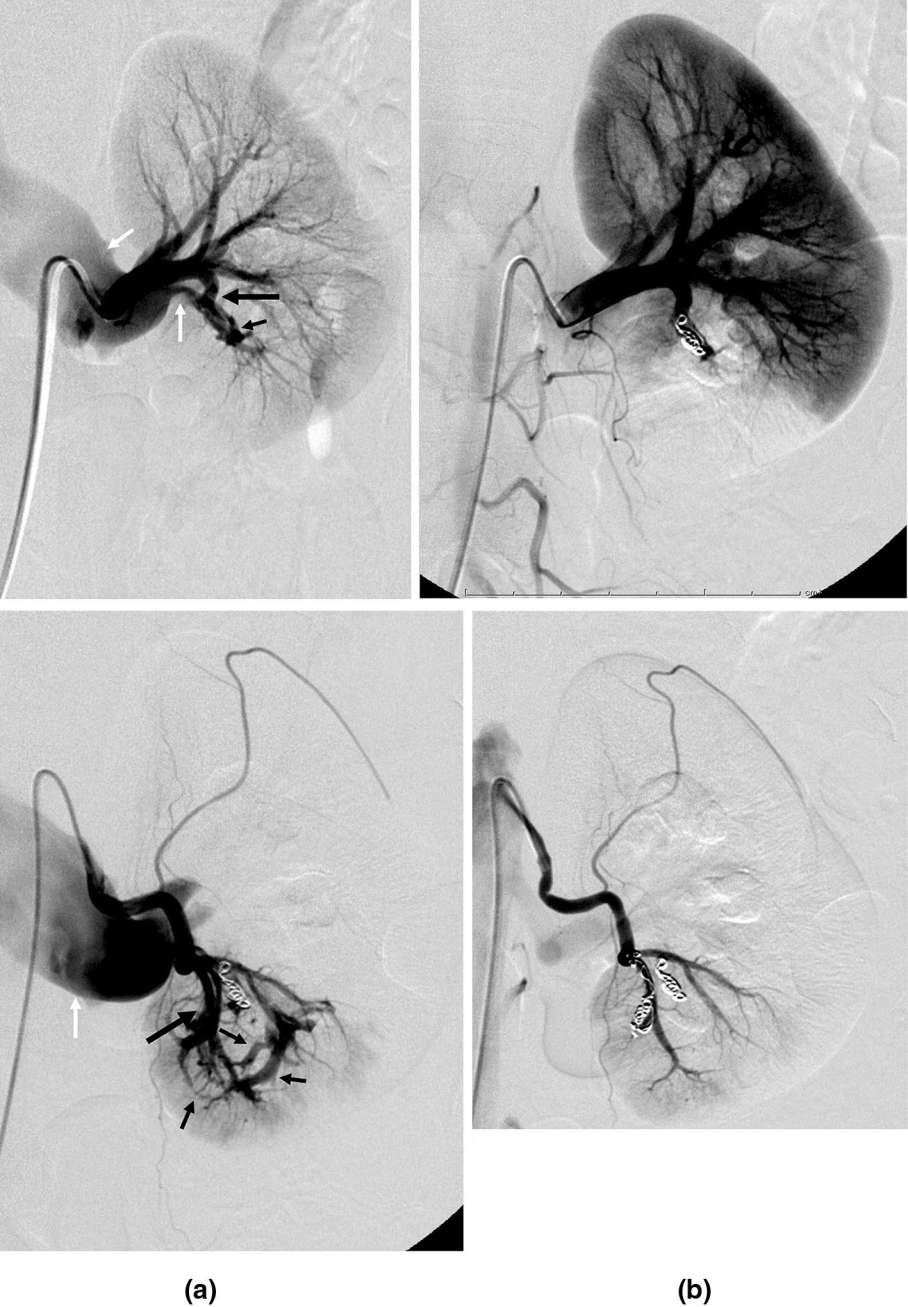
Angiography reveals a dilated renal vein draining a pseudoaneurysm on a lower pole branch of the main left renal artery (**1a**), as well as a dilated vein draining an arched pseudoaneurysm on another breach of the left renal artery (**2b**). White arrow: dilated draining vein, small black arrow: pseudoaneurysm, large black arrow: branch of the left renal artery. After TAE using microcoils (arrow), the dilated draining vein and pseudoaneurysm became undetectable (**1b**, **2b**). White arrow: dilated left renal vein, small black arrow: pseudoaneurysm or AVF, large black arrow: left renal artery and branches

Angiography revealed an arteriovenous fistula (AVF), with a dilated renal vein draining a pseudoaneurysm on a lower pole arterial branch of the main left renal artery (Fig. 2-1a). Transcatheter renal arterial embolization (TAE) was performed using 2 platinum microcoils (Tornado^®^, Cook Group Company) with a diameter of 0.018 in. and a length of 4–6 cm. After TAE, the dilated draining renal vein and pseudoaneurysm were no longer detected (Fig. 2-1b). However, another AVF was detected with a dilated vein draining an arched pseudoaneurysm on the second branch of the main left renal artery (Fig. 2-2a). TAE was again performed using 2 platinum microcoils, and the dilated draining vein and pseudoaneurysm became undetectable (Fig. 2-2b). After these two AVFs were embolized, the patient’s macroscopic hematuria subsided. Two weeks later, her Hb was increased to 12.8 g/dL without blood transfusion. Serum creatinine was 0.81 mg/dL even after TAE against AVF.

## Renal biopsy findings

**Fig. 3:**
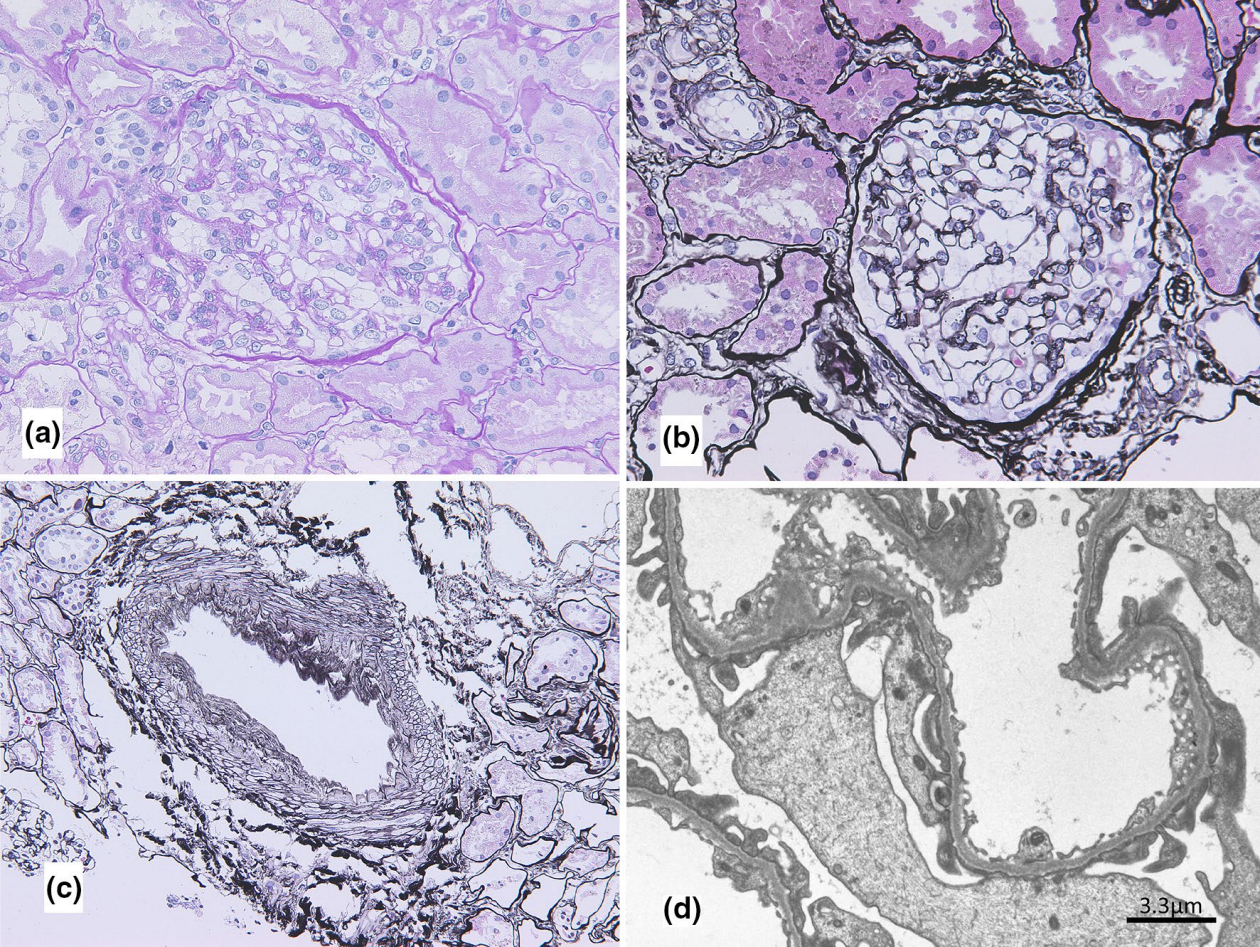
Histopathological tissue of the kidney biopsy. **a** PAS, **b** PAM-Masson, **c** PAM-Masson, **d** electron microscopy (thin basement membrane disease was diagnosed with the GBM thickness of 230 nm)

Histopathological examination of the biopsy specimen showed mild endothelial swelling without mesangial proliferation. The ratio of cortex and medulla was 6:4. Interlobular arteries of kidney were detected in the obtained pathological tissue. Immunofluorescence was negative for IgG and IgA, while IgM was weakly positive in the mesangium. However, C1q, C3, and C4 were all negative. Electron microscopy did not reveal any electron dense deposit. Thin basement membrane disease was diagnosed with the GBM thickness of 230 nm (Fig. 3).

## Discussion

On this patient, AVF formation of two renal arterial branches contributed to macrohematuria 5 days after percutaneous renal biopsy. AVF formation was diagnosed as definite using CT angiography instead of Doppler ultrasonography. She was treated with TAE. Late-onset renal bleeding may occur.

Korbet et al. evaluated the outcome of PRB on the native kidney in 1,055 adult patients (≥ 15 years). A 14-gauge Tru-cut needle was used for the initial 229 biopsies (1983–1990), while a 14-gauge automated biopsy needle was employed for the subsequent 826 biopsies (1991 to March 2012). The major complication rate was 5.7% during the first period and 6.9% in the second period. Baseline predictors of complications were a systolic blood pressure > 170 mmHg (odds ratio [OR] 4.2, 95% confidence interval [CI] 1.8–9.8), bleeding time > 7.5 min (OR 1.7, 95% CI 1.2–2.5), and serum creatinine > 3.5 mg/dL (OR 1.8, 95% CI 1.2–2.9) [1]. In addition, Simard-Meilleur et al. evaluated 312 biopsies performed between January 2007 and July 2011. The risk of symptomatic hematoma was strongly associated with platelet count, since it was 11% in patients with a platelet count > 200 × 10^9^/L, but, respectively, increased to 20%, 35%, and 40% as the platelet count declined to 140–200, 100–140, and < 100 × 10^9^/L (*p* = 0.00) [2]. A systematic review and meta-analysis about the risk of hemorrhagic complications associated with renal biopsy suggested that native kidney biopsy using automated biopsy devices and real-time ultrasonography is associated with a relatively small risk of macroscopic hematuria and erythrocyte transfusion requirement, and using smaller gauge needles may lower complication rates [4].

However, late-onset renal bleeding has occasionally been reported. Franke et al. reported one case of severe bleeding at 3 weeks after biopsy, with a decrease of hemoglobin by > 3 g/dL [5]. In addition, Murakami et al. reported the unexpected detection of a giant fistula by ultrasonography at 9 years after biopsy [6]. There have been a few reports on the technical success and effectiveness of embolization for post-biopsy AVF, including the study by Lorenzen et al., who found that embolization was successfully achieved without complications in 19 of 20 patients with AVF after biopsy of a transplanted kidney [7].

Renal AVF is an anomalous direct communication between the renal arteries and veins, and is usually an acquired lesion. Thus, AVF differs from a renal arteriovenous malformation (AVM), which is primarily a congenital abnormality. The majority of renal AVFs are iatrogenic and occur as a complication of renal biopsy or renal surgery, but these lesions can also be caused by blunt or penetrating trauma, inflammation, and malignancy [8].

The kidney biopsy for hematuria alone case is indicated for the further evaluation of genetic condition including Alport syndrome, because this patient had family history of renal disease [9].

In conclusion, we presented a Japanese patient with the onset of AVF-related macroscopic hematuria 5 days after PRB. This patient did not have any risk factor contributing to renal bleeding such as exercise, the use of anticoagulant, etc. Renal bleeding was effectively controlled by TAE. Severe renal bleeding resulting in urinary tract hematoma has usually been reported to occur within 24 h after renal biopsy, but the possibility of late-onset renal bleeding should be remembered.

## Abbreviations

AVM: Arteriovenous malformation
AVF: Arteriovenous fistula
CT: Computed tomography
TAE: Transcatheter arterial embolization
